# Development of the A-DIVA Scale: A Clinical Predictive Scale to Identify Difficult Intravenous Access in Adult Patients Based on Clinical Observations

**DOI:** 10.1097/MD.0000000000003428

**Published:** 2016-04-22

**Authors:** Fredericus H. J. van Loon, Lisette A. P. M. Puijn, Saskia Houterman, Arthur R. A. Bouwman

**Affiliations:** From the Department of Anesthesiology (FHJL, LAPMP, ARAB), Catharina Hospital Eindhoven, The Netherlands; Department of Research and Education (FHJL, SH), Catharina Hospital Eindhoven, The Netherlands; Fontys University of Applied Sciences (FHJL), Eindhoven, The Netherlands; Department of Signal Processing Systems and Electrical Enginereering (ARAB), TU/e University of Technology, Eindhoven, The Netherlands.

## Abstract

Placement of a peripheral intravenous catheter is a routine procedure in clinical practice, but failure of intravenous cannulation regularly occurs. An accurate and reliable predictive scale for difficult venous access creates the possibility to use other techniques in an earlier time frame.

We aimed to develop a predictive scale to identify adult patients with a difficult intravenous access prospectively: the A-DIVA scale.

This prospective, observational, cross-sectional cohort study was conducted between January 2014 and January 2015, and performed at the department of anesthesiology of the Catharina Hospital (Eindhoven, The Netherlands). Patients 18 years or older were eligible if scheduled for any surgical procedure, regardless ASA classification, demographics, and medical history.

Experienced and certified anesthesiologists and nurse anesthetists routinely obtained peripheral intravenous access. Cannulation was performed regarding standards for care.

A failed peripheral intravenous cannulation on the first attempt was the outcome of interest.

A population-based sample of 1063 patients was included. Failure of intravenous cannulation was observed in 182/1063 patients (17%). Five variables were associated with a failed first attempt of peripheral intravenous cannulation: palpability of the target vein (OR = 4.94, 95% CI [2.85–8.56]; *P* < 0.001), visibility of the target vein (OR = 3.63, 95% CI [2.09–6.32]; *P* < 0.001), a history of difficult peripheral intravenous cannulation (OR = 3.86, 95% CI [2.39–6.25]; *P* < 0.001), an unplanned indication for surgery (OR = 4.86, 95% CI [2.92–8.07]; *P* < 0.001), and the vein diameter of at most 2 millimeters (OR = 3.37, 95% CI [2.12–5.36]; *P* < 0.001). The scoring system was applied in 3 risk groups: 36/788 patients (5%) suffered from a failed first attempt in the low-risk group (A-DIVA score 0 or 1), whereas the medium (A-DIVA score 2 or 3) and high-risk group (A-DIVA score 4 plus), included 72/195 (37%) and 74/80 (93%) patients with a failed first attempt of inserting a peripheral intravenous catheter, respectively.

The additive 5-variable A-DIVA scale is a reliable predictive rule that implies the probability to identify patients with a difficult intravenous access prospectively.

Dutch Trial Register (ref: 4595).

## INTRODUCTION

Peripheral intravenous cannulation is the most common invasive procedure in clinical practice for administering fluids and medication. Among hospitalized patients, ∼70% to 80% need a peripheral intravenous catheter.^1–3^ However, as a straightforward and routine procedure, peripheral intravenous access is not easily obtained in all patients.^1,4–6^ This can be frustrating to medical professionals, but more importantly, it can be a painful, uncomfortable, and stressful experience for an already anxious patient, especially when multiple attempts are necessary.^6–8^ Moreover, multiple unsuccessful attempts to cannulate a peripheral vein create a time-consuming situation and are associated with additional risks as nerve damage, paresthesia, hematoma, and arterial puncture.^4^

Successful peripheral intravenous cannulation can be influenced by various factors, such as palpable or visual absence of a vein, as well as diabetes mellitus, sickle cell disease, body habitus, vascular pathology, physician in training, burn injuries, intravenous drug abuse, fluid status, sex, and age.^4,9–13^

In children, it is possible to predict the likelihood of a failure of intravenous cannulation on the first attempt by using the DIVA scale, a 4-variable proportionally weighted rule.^8,14^ Yen and colleagues reported the DIVA rule to be a useful aid in identifying children who may benefit from interventions that improve success rates of intravenous cannulation, but which are too resource-consuming to be used in all patients.^14^ In a subsequent study, Riker and colleagues concluded that the use of the DIVA scale can guide the implementation of adjunctive modalities to assist in obtaining timely vascular access, especially in those pediatric patients for who traditional techniques might contribute to increased pain and anxiety, dissatisfaction with received care and delay in treatment.^8^

A difficult intravenous access scale for adult patients (A-DIVA scale) is currently lacking. Such a scale could be used to prospectively identify patients with a high probability of a difficult intravenous access based on easily available clinical data, which may improve clinical practice and patient's comfort. In general, prediction models are developed to aid healthcare providers in estimating the probability or risk that a specific outcome is present in patients, and to inform their decision-making.^15^ When the inserter is not able to locate the target vein using palpitation or visualization, blind cannulation can be performed using landmarks and a trial and error approach. Alternatively, ultrasound provides a useful advanced technique. In addition, an accurate and reliable A-DIVA scale creates the possibility to use other techniques, such as ultrasound or the call for assistance of more-experienced individuals, in an earlier time frame.^16^

The aim of this study was to primarily identify risk factors for failure to perform peripheral intravenous cannulation on the first attempt in adult patients. Subsequently, the simplified additive A-DIVA scale creates a possibility to calculate the risk of failure during intravenous cannulation on the first attempt and to classify patients with a difficult intravenous access prospectively.

## METHODS

### Design

This prospective, observational, cross-sectional cohort study was conducted between January 2014 and January 2015.

### Setting

This study was performed at the Catharina Hospital (Eindhoven, The Netherlands), which is a 700-bed tertiary hospital specialized in cardiothoracic, bariatric, and oncological surgery.

The institutional review board (Catharina Hospital, Eindhoven, The Netherlands) approved the study protocol (ref: 2013-59) and the study protocol was registered in the Dutch Trial Register (ref: 4595). Written informed consent was obtained from all patients.

### Participants

Patients 18 years or older were eligible if scheduled for any surgical procedure, regardless of American Society of Anesthesiology (ASA) classification, demographics, and medical history. Patients were excluded if they did not understand or answer the questionnaire (due to physical or communicational disorders), were unresponsive or when intravenous access had been gained in the ward.

### Primary Outcome

The primary outcome variable was defined as failed peripheral intravenous cannulation on the first attempt. Peripheral intravenous cannulation was defined as successful, if a saline flush could be injected without signs of subcutaneous injection. An attempt was determined as the period between the needle first touched the skin until the needle was removed from the skin. After a failed attempt, a new attempt was stated to be any change in localizing a vein, followed by a new skin puncture.

### Predictors

Items included in the A-DIVA scale were selected from clinical observations, literature search in recent publications, and by expert opinions in a brainstorm discussion session consisting of anesthesiologists and nurse anesthetists. As a result of the brainstorm session, an agreement was reached according to factors, which have a possible relation with the primary outcome and should therefore be included in the study protocol. Recorded parameters included: patient's dominant side (left or right), received premedication, skin shade classified on a 3-point scale based on origin (Caucasian, Hispanic, or African American), diameter of the vein measured in millimeters with a ruler placed upon the vein after applying a tourniquet, whether or not the vein could be identified by palpating and/or visualizing the upper extremity, and if it was difficult to achieve an intravenous access in the past. After inserting the intravenous catheter, procedure-related data (size of the intravenous catheter, side of cannulation, place of cannulation on the extremity, pain score after cannulation on an 11-point NRS scale, number of attempts needed for successful intravenous cannulation, and the years of experience of the physician), demographic data (sex, ASA physical status, age, weight, length, body mass index, whether or not the patient fastened from oral foods and drinks for at least 6 hours preoperative, and if the patient was scheduled for elective or unplanned surgery) and patients medical history (chronic diseases, intravenous drug abuse, alcohol abuse, smoking, vessel diseases, a history of chemotherapy treatment, hematological status, the use of medications, and hypovolemia due to dehydration or treatment with hemodialysis) were collected by asking the patient or from the preoperative anesthesia screening form. Time needed to insert a peripheral intravenous catheter was registered from identifying the target vein until the intravenous catheter was secured after a successful attempt. The attending physician collected and recorded the data; completed register forms were analyzed and included in the dataset.

### Procedure

Experienced and certified anesthesiologists and nurse anesthetists, who gained at least 3 years of experience during training for certification and were familiar with the study protocol, routinely obtained peripheral intravenous access in the preoperative holding area. Intravenous catheters sizes 14 to 22 gauge, which were used in the hospital, were inserted (Venflon Pro Safety, BD Infusion Therapy AB, Helsingborg, Sweden). The size of the inserted catheter depended on the clinical situation; the size of the chosen catheter was determined by the expected surgical complexity, complications, and blood loss. Before cannulation, a tourniquet was placed on the upper extremity, at least 10 cm proximal to the elbow crease, to apply dilatation of the target vein.^17,18^ The tourniquet was tightened while maintaining pulsations of the radial artery. The skin was cleaned with chlorhexidine 70%. Palpating and visualizing the upper extremity helped identifying the target vein. Veins on the dorsal and ventral surfaces of the upper extremity were considered for peripheral cannulation, including the metacarpal, cephalic, basilic, and median veins. Intravenous cannulation was performed according to practice guidelines.^19,20^

### Sample Size

Based on the recent literature, we expected a failure of inserting a peripheral intravenous catheter on the first attempt in 15% of patients.^4,13^ For each predictor in the univariate logistic model, at least 5 patients with a present primary outcome needed to be enrolled in the study cohort. Finally, a minimum of 10 patients with an event for each predictor in the multivariate logistic model needed to be included. For this reason, we expected the need of a convenience and quota sampling of at least 1000 patients to be included in this study to ensure a balanced distribution across the desired variables.

### Statistical Analysis

To compare patients regarding the primary outcome, the chi-squared test, Fisher's exact test, Mann–Whitney *U*-test, and the unpaired sample *T*-test were performed as appropriate.

Potential risk factors were identified in a univariate logistic regression analysis. Items with a *P* > 0.10 were eliminated from the model. Significant associated items with the primary outcome from the univariate logistic model were entered in a multivariate logistic regression model. Variables were removed from this model using a backward elimination process, with the removal criteria set at *P* < 0.001. The definitive predictive scale was constructed by including significant variables from the multivariate logistic analysis. The effect size of all independent predictors was reported with adjusted odds ratios and 95% confidence intervals (CI). Collinearity between variables and the outcome of interest was identified with a logistic regression technique.

The additive A-DIVA scale was created by deriving ß coefficients from the logistic regression model. The additive points were calculated by taking the specific ß coefficient for each independent predictor, divided by the lowest ß coefficient of all the independent predictors, multiplied by 2, and rounded to the nearest integer. Each patient received an additive risk score based on the sum of the points of each predictor. Results of this additive score were used to define 3 risk groups (low, medium, and high risk).^21^

The overall fit (calibration) of the predictive scale was assessed using Hosmer–Lemeshow statistic. If the Hosmer–Lemeshow goodness-of-fit test statistic was >0.05, we failed to reject the null hypothesis that there was no difference between observed and model-predicted values, implying that the model fitted the data at an acceptable level.^22^ Analyzing the area under the curve (AUC) of the plotted receiver operating characteristics (ROC) curve represents the discriminative acquisition of the additive A-DIVA scale by assessing the ability to predict the risk of failure to insert a peripheral intravenous catheter.^23^

Bootstrapping resulted in stable and nearly unbiased estimates of performance.^15,24^ Bootstrap resampling started with fitting the logistic model in a bootstrap sample of 800 subjects, which was drawn with replacement from the original sample, 100% of the dataset was used for constructing and validating the A-DIVA scale. Averages of performance measures were taken >1600 repetitions.^15,24^

This multivariate prediction scale was reported according to the TRIPOD Statement.^15^ Throughout the study, a *P* < 0.05 was denoted as statistical significant. SPSS, version 21.0 (SPSS Inc, Chicago, IL) was used for all statistical analysis.

## RESULTS

Of the 1104 patients originally enrolled in this study, 41 patients were excluded for incomplete data. The data from the 1063 remaining patients were evaluated. Differences regarding patient's clinical characteristics were as shown in Table 1.

**TABLE 1:**
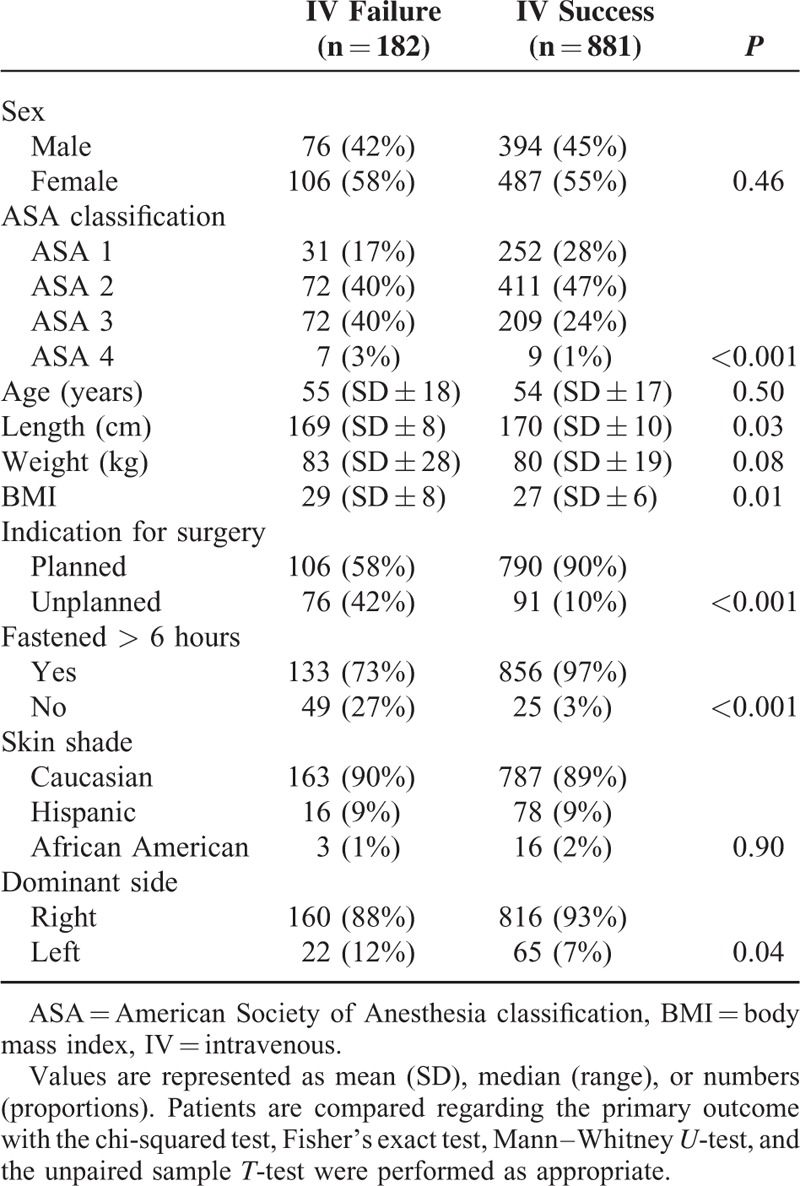
Baseline Clinical Characteristics of Patients With a Failed First Attempt of Intravenous Cannulation and With a Successful First Attempt

Data related to the procedure were outlined in Table 2. Failure to insert a peripheral intravenous catheter on the first attempt occurred in 182/1063 patients (17%). Two attempts were needed in 116 patients (11%), whereas 29 patients (3%) needed 3 attempts, 18 patients (2%) needed 4 attempts, and 19 patients (2%) needed 5 or more attempts to achieve a successful intravenous access. In this study cohort, a median number of 1 (range 1–8) attempts was required to insert an intravenous catheter successfully. The target vein was visible in 917/1063 patients (86%), identifying a vein by palpating the extremity was possible in 926/1063 patients (87%), and 84/1063 patients (8%) neither had a visual or palpable apparent vein. A known history of difficult peripheral intravenous cannulation was registered in 286/1063 patients (27%). The mean vein diameter was 2.3 mm (SD ± 1.1) in the group of patients with a failed first attempt of inserting a peripheral intravenous catheter, which was smaller compared to a mean vein diameter of 3.3 mm (SD ± 1.1) as obtained in the group of patients with a successful first attempt (*P* < 0.001). Patients with a successful first attempt reported a median pain score of 3 (range 0–8) on an 11-point NRS scale, whereas patients with a failed first attempt reported a median pain score of 6 (range 1–10) (*P* < 0.001).

**TABLE 2:**
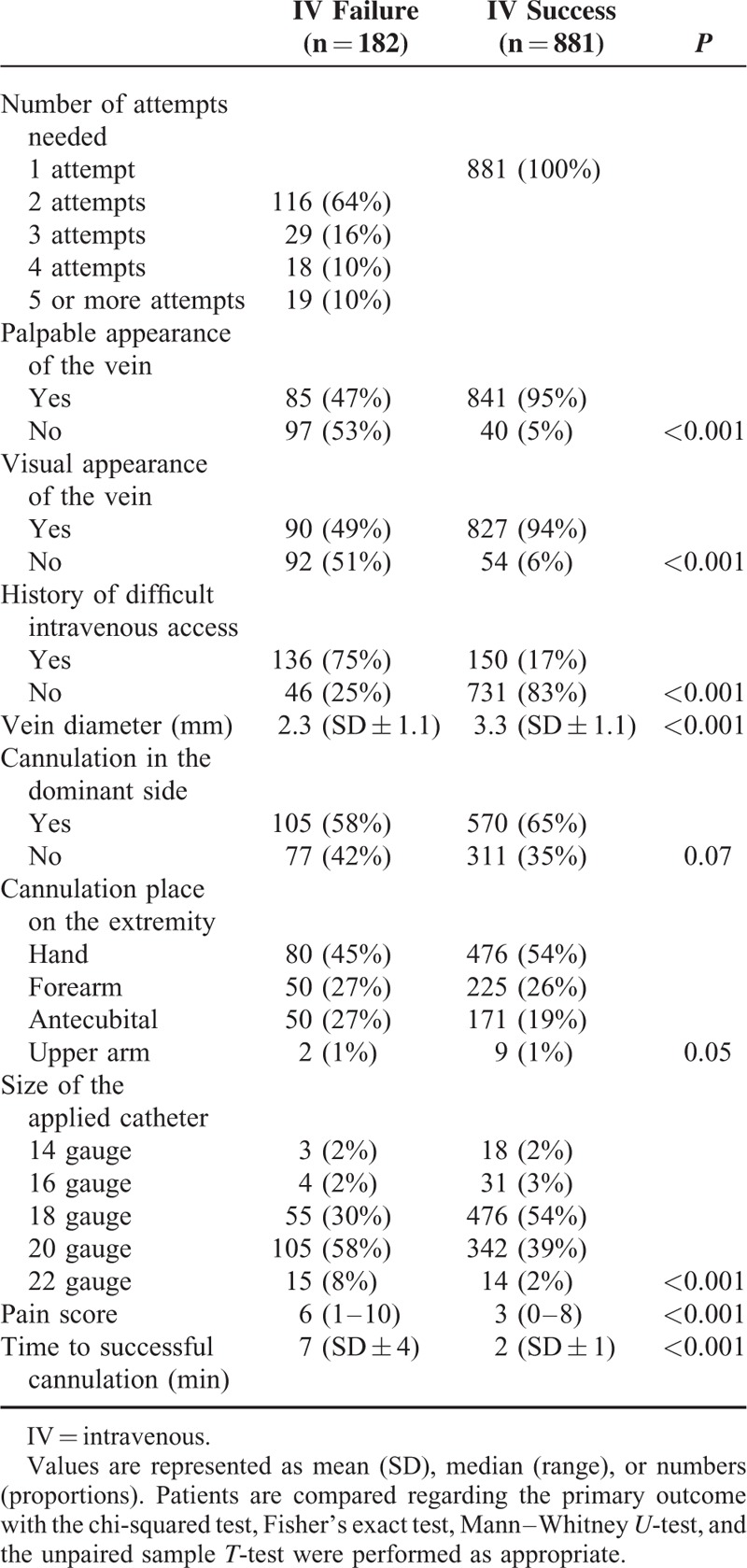
Data Related to the Procedure, in Patients With a Failed First attempt of Intravenous Cannulation and With a Successful First Attempt

The univariate logistic regression analysis identified 16 candidate variables (ASA classification, length, weight, BMI, an unplanned indication for surgery, preoperative fasting, palpability of a dilated vein, visibility of a dilatated vein, a known history of difficult peripheral intravenous cannulation, a dilated vein diameter smaller than 2 millimeters, cannulation in patient's dominant side, size of the used intravenous catheter, place of cannulation on the extremity, vascular diseases, preoperative hypovolemia, and renal insufficiency), as detailed in Table 3. These variables were afterward entered in a multivariate logistic regression analysis with failure on the first attempt of inserting a peripheral intravenous catheter as the primary outcome variable (Table 4). Palpability of the target vein (OR = 4.94, 95% CI [2.85–8.56]; *P* < 0.001), visibility of the target vein (OR = 3.63, 95% CI [2.09–6.32]; *P* < 0.001), a history of difficult peripheral intravenous cannulation (OR = 3.86, 95% CI [2.39–6.25]; *P* < 0.001), an unplanned indication for surgery (OR = 4.86, 95% CI [2.92–8.07]; *P* < 0.001), and a vein diameter of at most 2 mm (OR = 3.37, 95% CI [2.12–5.36]; *P* < 0.001) were associated with a failed cannulation on the first attempt as a result of the multivariate analysis.

**TABLE 3:**
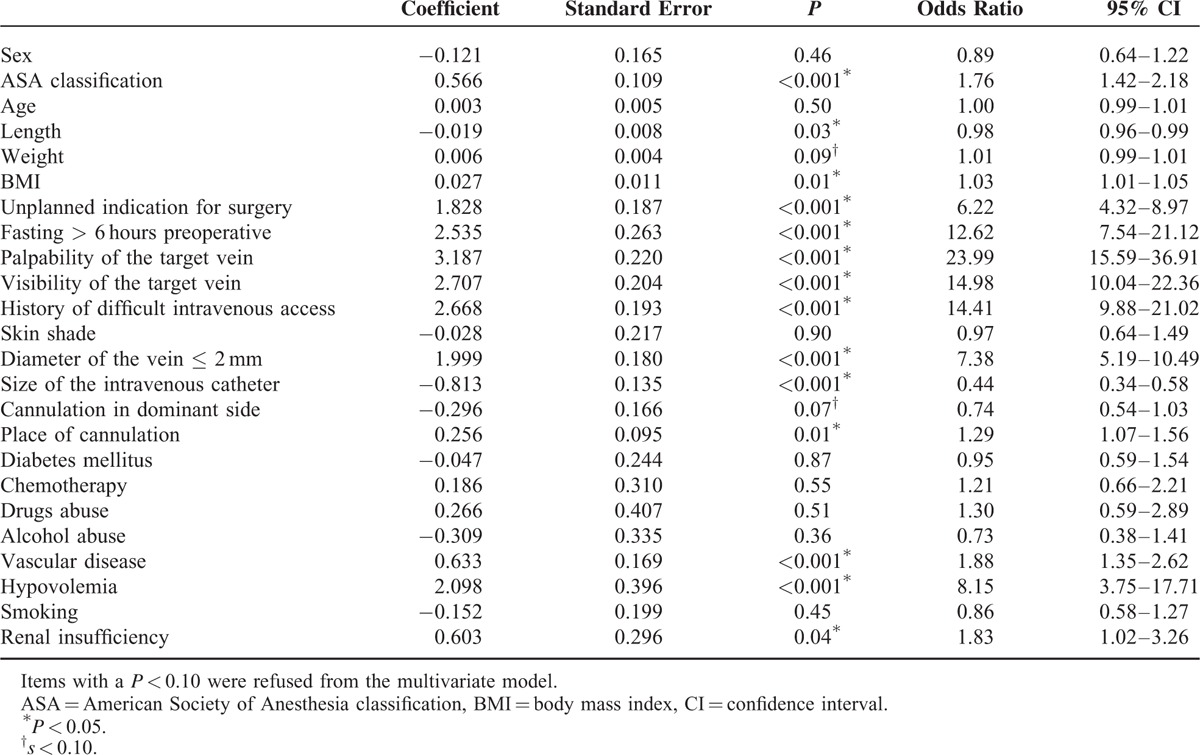
Univariate Logistic Regression Analysis, Identifying Potential Risk Factors Which are Associated With a Failed First Attempt of Peripheral Intravenous Cannulation

**TABLE 4:**
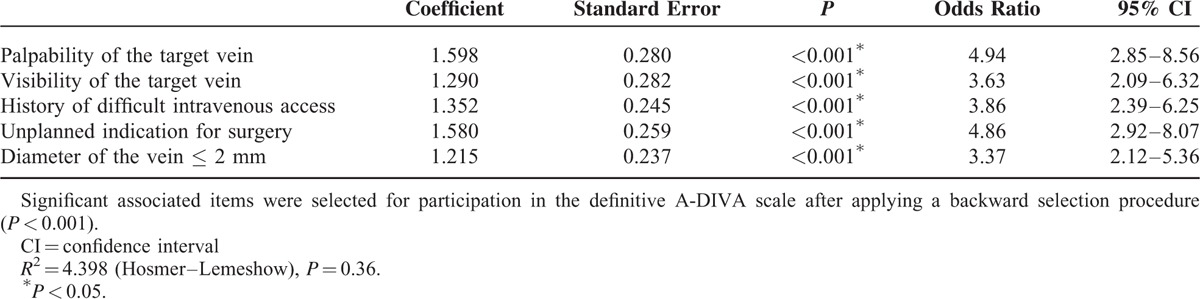
Multivariate Logistic Regression Analysis

An interaction was detected between palpability and visibility of the vein. When both risk factors were present in a patient, the odds ratio increased to 42.71 (95% CI [22.90–79.66]; *P* < 0.001). No collinearity could be identified between the size of the intravenous catheter and the diameter of the vein on the outcome of interest. Even years of experience of the depending physician did not show an interaction with failure of peripheral intravenous catheter placement on the first attempt.

The simplified additive A-DIVA scale was derived from the ß coefficients for each variable and was represented in Table 5. All factors included in the A-DIVA scale had a comparable value for each additive risk factor and were therefore rounded to 1. The scores for existing risk factors represented an approximate percentage of a predicted difficult intravenous access for each patient. When the scoring system was applied to all patients, 3 different risk groups were created. The low risk group (A-DIVA score 0 or 1) included 36/788 patients (5%) with a failed first attempt of inserting a peripheral intravenous catheter. In the medium risk group (A-DIVA score 2 or 3) and high-risk group (A-DIVA score 4 plus), 72/195 (37%) and 74/80 (93%) patients suffered from a failed first attempt respectively (Table 6).

**TABLE 5:**
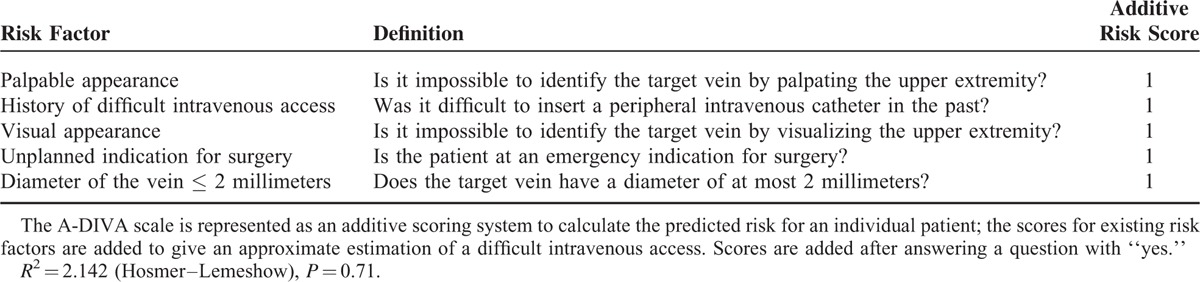
Risk Factors, Definition, and the Additive Score

**TABLE 6:**
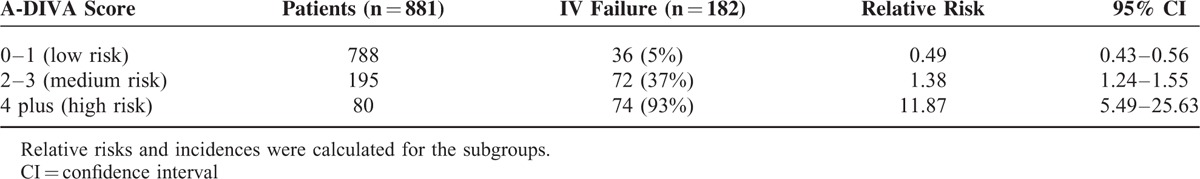
Application of the A-DIVA Scale, With Patients Allocated to 1 of the 3 Subgroups (Low, Medium, or High Risk) Regarding Their Individual Score on the Additive A-DIVA Scale

**FIGURE 1:**
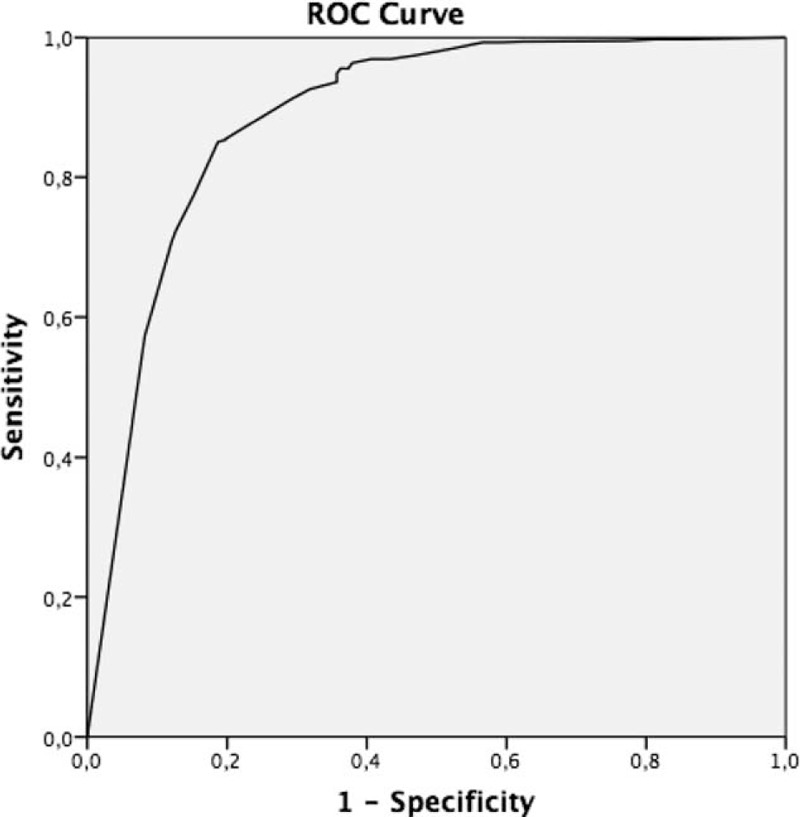
Receiver operating characteristics (ROC) curves for the simplified additive A-DIVA scale. A-DIVA = Adult Difficult Intravenous Access.

The ROC curve of the additive A-DIVA scale showed an AUC of 89% (SE = 0.016) and was represented in Figure 1. Goodness of fit of the additive A-DIVA scale, tested with the Hosmer–Lemeshow statistic, resulted in a *R*^2^ value of 2.142 (*P* = 0.71).

## DISCUSSION

In this prospective, observational cohort study, we identified 5 risk factors, which were associated with a failed first attempt of inserting an intravenous cannulation. These 5 risk factors (e.g., palpability of the target vein, visibility of the target vein, difficult peripheral intravenous cannulation in patient's history, an unplanned indication for surgery, and a vein diameter of at most 2 mm) were included in the A-DIVA scale for its use as a clinically applicable predictive rule in daily anesthesia practice. A score on the A-DIVA scale, however, will predict the likelihood of failed peripheral intravenous catheter placement in a group of patients with a similar risk profile. In fact, a higher score on the A-DIVA scale indicates a higher risk for difficult intravenous catheter placement.

Difficult intravenous access is a frequently encountered clinical challenge, which has been subject of research in various previous publications.^25–28^ Reported success rates of first attempt peripheral intravenous cannulation varies from 98% to as low as 51%, whereas our study shows an 83% success rate upon inserting a peripheral intravenous catheter on the first attempt.^4,13,25–28^

In the recent literature, many risk factors for a difficult intravenous access have been identified. Our data shows the risk of failed intravenous catheter placement increased substantially if visual identification and/or if identifying the target vein by palpating the extremity was impossible, which is in line with the observations of Guillon and colleagues.^29^ However, blind insertion based on landmarks in a trial-and-error technique is no longer justified as technical resources, such as ultrasound, are widely available to support venous access. Another important risk factor for a difficult intravenous access was a failed first attempt in the patient's history, which indicates patients to be at a 4-fold increased risk for a failed first attempt of peripheral intravenous cannulation in future attempts. Although this seems trivial, previous studies did not establish patient's history to be a risk factor for a difficult intravenous access. To continue, an unplanned indication for surgery even acts as a risk factor in the A-DIVA scale, which is in line with observations of previous studies.^10,30,31^ In our dataset, patients who were admitted for any type of nonelective surgery were designated as indicated for unplanned surgery. Logically, polytrauma patients and patients in shock are at an increased risk for a difficult intravenous access due to hypotension and/or hypovolemia. The reason for a smaller vein diameter to act as a risk factor in our predictive model may be explained by the difficulty to identify the target vein by palpating or visualizing the extremity.

A predictive scale has to be effective and efficient in its use. For this reason, a compromise had to be reached so that the A-DIVA scale recognizes patients at increased risk with an optimal level of discriminative acquisition, but remains simple enough with the smallest set of risk factors to be used in daily clinical practice.^32^ The Hosmer–Lemeshow statistic resulted in a minimum set of 5 risk factors to develop the predictive scale. Clinical usability was improved by constructing an additive scoring system, which resulted in the 5-variable additive A-DIVA scale. This scale shows to have an optimal level of discriminative acquisition with an area under the ROC curve of 89%. The AUC is widely recognized as the measure of a diagnostic test's discriminatory power and indicates the probability that a random pair of test results will be ranked correctly, whereas discrimination refers to the ability to distinguish high-risk patients from low-risk patients. In addition, internal validation refers to the performance in patients from a similar population as where the sample originated from.^24^ The most efficient internal validation has been claimed to be achieved by the bootstrapping technique, which replicates the process of sample generation from an underlying population by drawing samples with replacement from the original dataset.^24,32^

An individual patient will either have a successful or failed first attempt of inserting a peripheral intravenous catheter, but no scoring system will precisely predict the outcome for a patient. Though, risk stratification helps eliminating bias against patients at high-risk for difficult intravenous access and may reduce complications related to the procedure.^15,33^ Nevertheless, the routine and straightforward nature of the procedure of inserting an intravenous catheter may imply that successful cannulation is likewise aphoristic, intravenous access is not easily obtained in all patients.^10^ To add on this, failure to obtain a peripheral intravenous access can delay diagnoses and treatment, and may expose patients to risks associated with central venous cannulation.^9^ Moreover, we believe that early recognition of patients at risk could help in applying alternative approaches, such as ultrasound guidance during catheter placement, to achieve a successful peripheral intravenous access. The use of venous access devices can be optimized through the implementation of the A-DIVA scale in daily practice by the identification of predictors of difficulty prospectively.^13,29^ As concluded by Liu and colleagues, the greatest success rate of ultrasound-guided placement of peripheral intravenous catheters was found in patients with a difficult intravenous access, especially in those whose veins were neither visible nor palpable.^34^ In general, we believe it would not improve efficacy nor be cost-efficient to apply new infrared and other devices in all patients. Furthermore, the proposed A-DIVA score may also be valuable in the evaluation of (cost-) efficacy and validation of the many venous access devices available in the market.

Our aim was to develop a simplified additive scale to predict the risk of a difficult intravenous access in adult patients. Yen and colleagues previously developed the 4-variable DIVA scale to identify children with a difficult intravenous access based on clinical observations. The DIVA scale was constructed with the variables age, a history of prematurity, vein visibility, and vein palpability. Some variables are only applicable to children because of specific anatomical characteristics in children and/ or differences in behavioral factors, although a visual and palpable absent vein were also included in our predictive scale as a risk factor for a difficult intravenous access.^14^

Several limitations have to be taken into account with interpretation of our results. Most importantly, external validation of the A-DIVA scale was not performed in the present study. The A-DIVA scale was developed in a cohort of patients scheduled for any surgical procedure, whereas experienced anesthesiologists and nurse anesthetists inserted the peripheral intravenous catheters in the preoperative holding area of our operation theatres. Internal validation was performed with a bootstrapping strategy. Further, internal validity may be seen as an approximation to external validity.^24^ To improve clinical usability and to confirm the A-DIVA scale to be generalizable to all hospitalized patients, it is essential to evaluate the performance of the scale in an external cohort. To continue, testing the A-DIVA scale in a new population will have to reveal changes in the performance characteristics of this scale when applied to a different group of patients in different departments of a hospital or by different nursing personnel. In summary, to be considered useful, a prediction model should be clinically credible, accurate (well calibrated with good discriminative ability), have generality (be externally validated), and provide useful information to clinicians that improve therapeutic decision-making and thus patient outcome.^24–36^

In conclusion, the 5-variable additive A-DIVA scale is a reliable and accurate predictive rule that implies the probability to identify patients with a difficult intravenous access. Applying the A-DIVA scale to surgical patients may increase the success rate of inserting a peripheral intravenous catheter on the first attempt. Otherwise, it creates a possibility to use other techniques, such as ultrasound, in an earlier time frame.
